# Suicide gene therapy by canine mesenchymal stem cell transduced with thymidine kinase in a u-87 glioblastoma murine model: Secretory profile and antitumor activity

**DOI:** 10.1371/journal.pone.0264001

**Published:** 2022-02-15

**Authors:** Antonio J. Villatoro, Cristina Alcoholado, María del Carmen Martín-Astorga, Nuria Rubio, Jerónimo Blanco, Cristina Pilar Garrido, José Becerra

**Affiliations:** 1 Laboratory of Bioengineering and Tissue Regeneration (LABRET), Department of Cell Biology, Genetics and Physiology, Faculty of Sciences, University of Málaga, IBIMA, Málaga, Spain; 2 Networking Research Center on Bioengineering, Biomaterials, and Nanomedicine (CIBER-BBN), Madrid, Spain; 3 Cell Therapy Group, Catalonian Institute for Advanced Chemistry (IQAC-CSIC), Barcelona, Spain; 4 Andalusian Centre for Nanomedicine and Biotechnology-BIONAND, Málaga, Spain; Università degli Studi della Campania, ITALY

## Abstract

The role played by certain domestic species such as dogs as a translational model in comparative oncology shows great interest to develop new therapeutic strategies in brain tumors. Gliomas are a therapeutic challenge that represents the most common form of malignant primary brain tumors in humans and the second most common form in dogs. Gene-directed enzyme/prodrug therapy using adipose mesenchymal stem cells (Ad-MSCs) expressing the herpes simplex virus thymidine kinase (TK) has proven to be a promising alternative in glioblastoma therapy, through its capacity to migrate and home to the tumor and delivering local cytotoxicity avoiding other systemic administration. In this study, we demonstrate the possibility for canine Ad-MSCs (cAd-MSCs) to be genetically engineered efficiently with a lentiviral vector to express TK (TK-cAd-MSCs) and in combination with ganciclovir (GCV) prodrug demonstrated its potential antitumor efficacy in vitro and in vivo in a mice model with the human glioblastoma cell line U87. TK-cAd-MSCs maintained cell proliferation, karyotype stability, and MSCs phenotype. Genetic modification significantly affects its secretory profile, both the analyzed soluble factors and exosomes. TK-cAd-MSCs showed a high secretory profile of some active antitumor immune response cytokines and a threefold increase in the amount of secreted exosomes, with changes in their protein cargo. We also found that the prodrug protein is not released directly into the culture medium by TK-cAd-MSCs. We believe that our work provides new perspectives for glioblastoma gene therapy in dogs and a better understanding of this therapy in view of its possible implantation in humans.

## Introduction

In recent years there has been enormous interest in the role played by certain domestic species such as dogs as a translational model in comparative oncology to develop new therapeutic strategies for brain tumors. The advantage of this model is based on the natural suffering of various types of cancer and the similarity with the human species of different environmental conditions, diet, as well as their evolution and responses to treatment, among others [1].

Cell-based gene therapy has shown interesting experimental results against cancer [2]. Among the different Trojan Horses used, mesenchymal stem cells (MSCs) are very interesting vehicles for the supply of antitumor agents due to their specific tropism towards the tumor, being the most used for suicide gene therapy [3].

This strategy involves the introduction of a sequence of genetic material in tumor cells, triggering the necessary mechanisms to activate the apoptotic pathways themselves or facilitate the activation of certain compounds that can spread to neighboring malignant cells and mediate their destruction [4]. Suicidal genes promote a cytotoxic effect after their expression in the target cell, by encoding enzymes that convert non-toxic prodrugs into highly toxic metabolites [5].

Adipose tissue mesenchymal stem cells (Ad-MSCs) genetically engineered to express the herpes simplex virus thymidine kinase (TK) in combination with the administration of ganciclovir (GCV) prodrug are a potentially effective treatment in different types of human tumors, highlighting glioblastoma [6, 7] having been employed in I/II clinical trials on gastrointestinal tumors [8]. TK catalyzes the phosphorylation of GCV, transforming it into the cytotoxic GCV tri-phosphorylated metabolite (pGCV), a nucleoside analogue. The incorporation of the pGCV in nascent DNA of proliferating cells results in chain termination and DNA polymerase inhibition leading to cell death by apoptosis [9]. *In vivo*, these cytotoxic cells are able to migrate to neoplasia, integrate into the tumor microenvironment shaped like blood vessels and exert antitumor therapy [10]. This action, known as the bystander effect, involves the transfer of phosphorylated toxic GCV and it has been attributed to different possible pathways, such as gap junctions [11, 12], inhibition of autophagy [13], apoptotic vesicles [14], and immunostimulatory paracrine effect [15], although this mechanism is not fully elucidated.

The safety and clinical efficacy of canine adipose tissue mesenchymal stem cells (cAd-MSCs) in the treatment of different pathologies in the canine species [16, 17] have been demonstrated. However, although different new strategies have been described in the treatment of canine tumors in recent years [18–20], cAd-MSCs have never been used as vehicles for TK gene therapy as suicide gene.

To date, the modifications introduced by the transduction of these cells in terms of their secretory profile and their anti-inflammatory and immunomodulatory capacities to investigate the mechanism involved in the MSCs as Trojan Horse have not been described either.

The aim of our study was to develop and characterize for the first-time therapeutic TK-expressing canine Ad-MSCs (TK-cAd-MSCs), evaluating their secretory profile, and demonstrating their antitumor efficacy *in vitro* and *in vivo* in a mice model with the glioblastoma cell line U87. Our results showed that TK-cAd-MSCs have antitumor efficacy on the U87 glioblastoma cells in vitro and in vivo and a change in their secretory profile, significantly increasing both the presence of certain molecules analyzed in the secretome and the production of exosomes that modify the proteomic profile of its cargo.

## Material and methods

### Animals

Eight adults severe combined immunodeficiency (SCID) mice (Harlan Laboratories) from 6 to 8 weeks of age were used and kept under the absence of pathogens in laminar flow boxes. Animal maintenance and experiments were performed in accordance with established guidelines of the Catalan Government and protocol num. 4565 approved by Direcció General del Medi Natural, Generalitat de Catalunya. Animal housing provided a 12 h light–12 h dark cycle, constant temperature of 26–27°C, and relative humidity of 45–60%. Mice were fed with a commercial laboratory animal diet for severe combined immunodeficiency (SCID) mice and water *ad libitum*. Animals were anesthetized with intraperitoneal injections of 0.465 mg/kg of xylazine and 1.395 mg/kg of ketamine before cell implantation and received buprenorphine in the drinking water after the intervention to minimize suffering.

### Cell culture

cAd-MSCs were isolated from the gluteal subcutaneous fat of three healthy donor dogs, as previously described [16, 17]. cAd-MSCs [21] and human glioblastoma U87 (HTB-14; ATCC) cells were cultured with Dulbecco´s modified Eagle´s medium (DMEM) containing 10% inactivated fetal bovine serum (FBS), 2.5 mM L-glutamine, 100U/mL penicillin, 100 μg/mL streptomycin, and 1.25 μg/mL fungizone (all Sigma-Aldrich). Cells were detached when confluence was over 80% and subcultured at a concentration of 10^4^ cells/cm^2^ for subsequent passaging. All cAd-MSCs experiments were performed at passage 3.

### Cell transduction

cAd-MSCs were labeled with CMV:hRluc:mRFP:tTK trifunctional lentiviral construct (TK-cAd-MSCs); and U87 cells with CMV:Pluc:eGFP; (PG-U87), as described before [10]. Viral particles were produced using Hek-293T/17 cells (ATCC, CRL-11268), viral envelope plasmid pCMV-VSVG (Addgene) and packaging construct pCMV-dR8.2 dvrp (Addgene) according to the manufacturers of second-generation lentiviral systems. The supernatant containing viruses was used to transduce cells by incubating with 8 μg mL^-1^ polybrene (Sigma-Aldrich). After 48 hours, transduced cells were sorted using FACSAria™ III (BD Biosciences).

### *In vitro* bioluminescence imaging (BLI) and analyses

BLI was performed using a high-efficiency ImagE-MX2 Hamamatsu-Photonics system provided with an EM-CCD digital camera, cooled at –80°C. Coelenterazine (PJK) was solubilized in NanoFuel solvent following the commercial protocol (3.33 mg mL^-1^) and stored at −80°C. For *in vitro* BLI, the medium was removed, washed with PBS, and imaged immediately following the addition of coelenterazine (0.1 mg mL^-1^). Light events were calculated using the Hokawo 2.6 image analysis software from Hamamatsu-Photonics Deutschland GmbH and expressed as photon counts (PHCs) after subtracting the background. Arbitrary colors representing light intensity levels were used to generate color images [6, 10].

### MTS cell proliferation assay

Cell doubling time (DT) was calculated using the formula DT = (t − t_0_) · log2 / log (N − N_0_), where t − t_0_ is culture time (h), N is the number of harvested cells, and N_0_ is the number of cells at the beginning [16]. Cell proliferation was measured using MTS assay (CellTiter 96 Aqueous One Solution Cell Proliferation Assay, Promega) [16, 22]. In a 96-well plate, 3000 cells/well were seeded and supernatants were collected on days 1, 2, 5, 7, 9, 12, 14 and 16 (n = 3/day, each condition). MTS reagent was added and absorbance was measured at 490 nm using a microplate reader (ELx800, Bio-Tek instruments).

### Flow cytometry

Fluorescence-activated cell sorting was used to characterize both cell types as previously described [16] against CD44, CD90, CD34, and CD105 (Miltenyi Biotec) using Gallios Flow Cytometer (Beckman Coulter).

### Karyotype

cAd-MSCs and TK-cAd-MSCs were karyotyped as previously described [16, 22]. Cells were cultured until semi-confluence, treated with colcemid (Thermo Fisher Scientific), stained with 2% Giemsa (Merck), and analyzed with ordinary bright-field microscopy.

### ELISA secretory profile assay

The secretory profile was determined from conditioned mediums (CMs) of cAd-MSCs and TK-cAd-MSCs in standard culture conditions. When confluence was achieved, CMs were collected, filtered (0.20 μm filter), and stored at −80°C. Cells were counted and viability was evaluated with trypan blue and the results were later normalized. Concentrations (pg/10^6^ cells) of 11 analytes were determined: chemokine (Monocyte Chemoattractant Protein-1, MCP-1); cytokines (Interleukins: IL-2, IL-6, IL-8, IL-10, IL-12p40, Tumor Necrosis Factor alpha: TNF-α, Interferon gamma: IFN-γ); and growth factors (Beta-nerve growth factor: NGF-β, Stem Cell Factor: SCF, Vascular Endothelial growth factor A: VEGF-A) using commercial Luminex kit canine cytokine 11-plex assay (Thermo Fisher Scientific) according to the manufacturers’ instructions [21].

### Indoleamine 2,3−dioxygenase (IDO) enzymatic activity

IDO enzymatic activity was determined by measuring spectrophotometrically its metabolite, kynurenine, as previously described [21]. CMs from cAd-MSCs and TK-cAd-MSCs, previously supplemented with L-Trp (Sigma Aldrich), were briefly incubated with 30% trichloroacetic acid (Sigma Aldrich) for 30 minutes at 50°C and centrifuged at 10000×g for 5 minutes. Then, the supernatant was mixed with Ehrlich’s reagent (Sigma Aldrich) in a 96 well plate and the optical density was measured at 490 nm in a microplate reader (ELx800, Bio-Tek instruments).

### Nitric oxide (NO) production

The amounts of NO metabolites (NO^2-^ and NO^3-^) produced in conditioned mediums were assessed using a photometric endpoint determination method (Nitrite/Nitrate colorimetric assay kit. Roche), as described by the manufacturers’ instructions [21].

### Exosomes isolation and characterization

cAd-MSCs and TK-cAd-MSCs exosomes were isolated by ultracentrifugation using 70 Ti rotor in an Optima LE-80K ultracentrifuge (Beckman Coulter) and characterized by transmission electron microscopy (TEM; Morgagni 268D electron microscope, Philips), Zetasizer Nano ZS (Malvern-Instruments), western-blot detection, and proteomic analysis by mass spectrometry were also carried out, as previously described [22]. Exosome quantification was performed using a Bicinchoninic acid kit (BCA; Thermo-Scientific).

### Modulation of canine peripheral blood mononuclear cells (cPBMCs) proliferation

cPBMCs from a healthy donor dog were separated using Ficoll-Hypaque density gradient centrifugation, stained with 4 μM 5-chloromethylfluorescein diacetate (CMFDA, Cell Tracker Green Kit C2925, Thermo Fisher Scientific, Inc.), and plated in a 96-well plate at a concentration of 5 × 10^4^ cells/well. cAD-MSCs and TK-cAd-MSCs, previously inactivated with mitomycin C for 3 hours, were seeded in the plate at 1 x 10^4^ cells/well. Concanavalin A (ConA; Sigma- Aldrich) was added to the experimental wells at a final concentration of 5 μg/ml, the amount of lymphocyte proliferation was analyzed after 72 hours by flow cytometer (Beckman Coulter). For comparison, lymphocytes stimulated with ConA were set to 100% proliferation. Flow cytometry data were analyzed using FlowJo cytometry software [17, 22].

### Antitumoral co-culture assays

To evaluate the antitumoral activity, TK-cAd-MSCs were co-cultured with tumoral glioblastoma PG-U87, previously tested in some of our studies [7, 10, 23]. Both cell types were seeded in co-culture in 96-well plates at a 1:1 cell ratio. Half of the samples received GCV (0.004 μg/μL) (Cymevene; Roche). Every 2 days, the medium was replaced with a fresh medium. Triplicate samples of each co-culture condition were analyzed by BLI on days 1 (before starting GCV treatment), 5, 7, and 9. On day 9, cells were also visualized by fluorescent microscopy. The experiment was repeated three times.

### *In vivo* mice glioblastoma model bystander cell therapy

The animals were anesthetized with intraperitoneal injections of 0.465 mg/kg of xylazine (Rompum, Bayer) and 1.395 mg/kg of ketamine (Imalgene, Merial Laboratories). For cell implantation, mice were mounted in a stereotactic frame (Stoelting) and secured. An intracranial drill hole was accomplished at 0.6 mm posterior (x-axis) and 2 mm lateral (y-axis) relative to bregma, and the cell suspension of tumor cells was injected in the injury site at a rate of 1.5 ml/min using a Hamilton syringe (700 series) at a depth of 2.75 mm (z-axis). The needle was removed slowly after waiting an additional 4 minutes. The scalp was closed with suture and animals received buprenorphine (Buprecare, Divasa-Farmavic) (0.9 mg/ml) in the drinking water. Mice (n = 8) were injected with 6x10^4^ glioblastoma PG-U87 cells. Six days later, the experimental group (n = 4) was inoculated with 8x10^6^ TK-cAd-MSC in the same way that tumor cells and the rest were left as control (n = 4). Four days later, all animals received daily intraperitoneal GCV (50 mg/kg) for three weeks. Animals received buprenorphine (Buprecare, Divasa-Farmavic) (0.9 mg/ml) in the drinking water after the intervention to minimize suffering. Animal health and behaviour were monitored every day. Tumor progression was analyzed weekly by BLI to study TK-cAd-MSC behavior *in vivo*. Tumor size was qualitatively measured by changes in light production, so plots of light emitted by PG-U87 cells showed tumor growth. Animals were anesthetized and sacrificed by cervical dislocation when signs of disease were noted. Experiments were performed by qualified staff with previous experience in this type of procedure, so no special training was required.

### *In vivo* bioluminescence imaging (BLI)

BLI image acquisition was done using a high-efficiency ImagE-MX2 Hamamatsu-Photonics system provided with an EM-CCD digital camera cooled at −80°C. D-luciferin (Regis Technologies) stock solution was prepared at a concentration of 16.5 mg/mL in PBS and stored at −20°C. For *in vivo* imaging anesthetized mice were inoculated intraperitoneally (i.p.) with 150 μL of luciferin (16.7 mg/mL) and imaged 15 minutes after substrate injection.

### Statistical analysis

Statistical analysis was performed by SigmaPlot 11.0. T-test was applied for 2 group comparisons and ANOVA test with Bonferroni post-test for more than 2 groups (normal distribution). Non-parametric two-sample Wilcoxon rank-sum (U-Mann-Whitney) test was applied for comparisons between 2 groups and Kruskal-Wallis with Tukey post-test for more than 2 groups. Statistically significant differences were considered when *P*-value < 0.05.

## Results

### Transduction of cAd-MSCs, BLI analysis and TK cytotoxic activity

**Fig 1:**
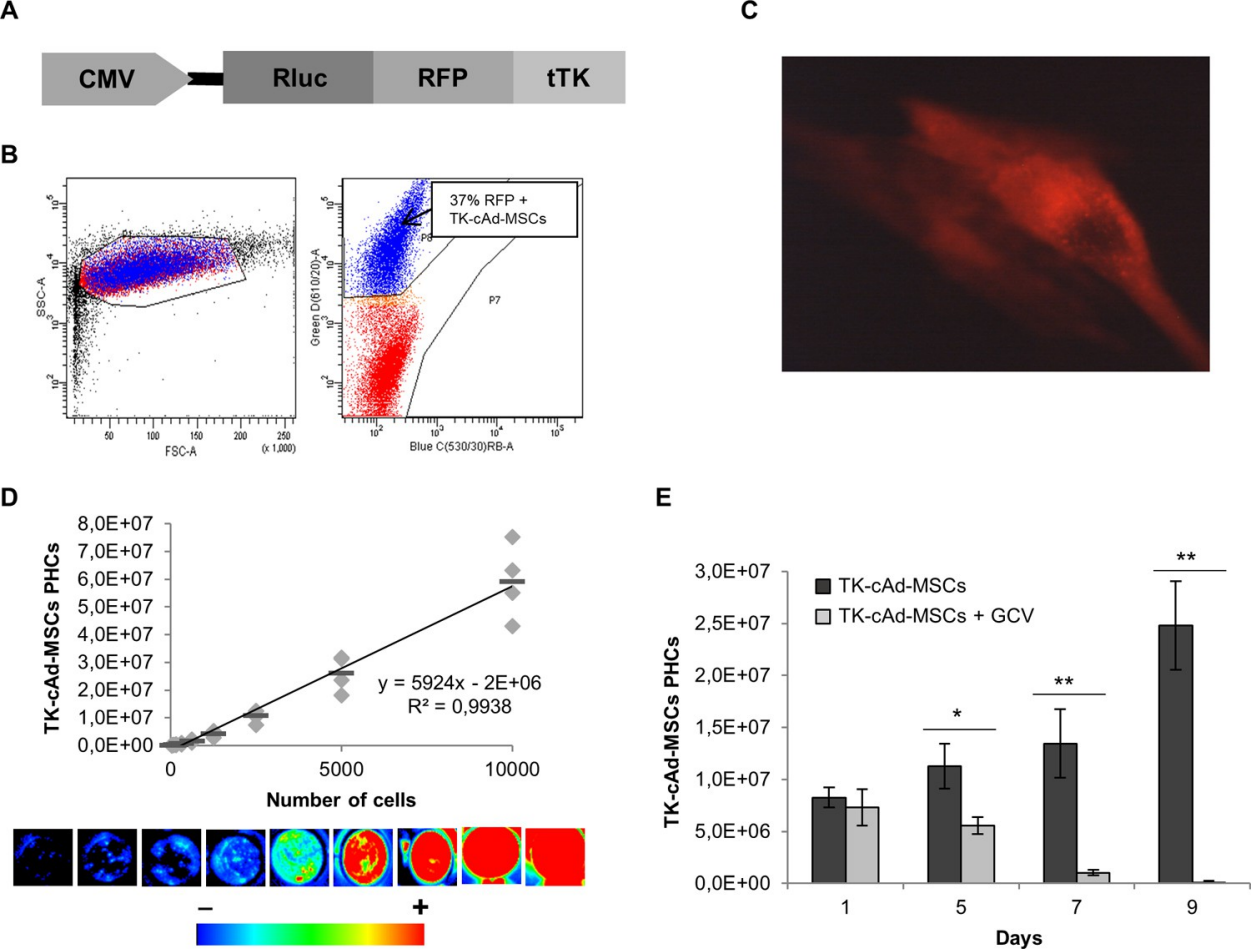
TK-cAd-MSCs sorting by RFP fluorescence, analyses for Rluc-BLI and the cytotoxic effect. (A) Schematic representation of CMV:hRluc:mRFP:tTK gene construction in lentiviral particles. (B) Histogram dot plot showing cell sorting by RFP positive fluorescence of transduced TK-cAd-MSCs (37% of RFP positive cells). (C) Fluorescent microscopy image of TK-cAd-MSCs 7 days after sorting (in red). The microscope image was taken with a Nikon eclipse ts100 microscope, 20× objective. (D) Linear correlation between Rluc PHCs and the number of TK-cAd-MSCs (n = 3). The equation and the R^2^ value of the trendline are shown. Insets; Composite pseudo-color representative BLI images that correspond to the serial dilutions are shown below the graph. Arbitrary rainbow color scale depicts light intensity (red: highest; blue: lowest) in BLI images. (E) Histograms comparing the *in vitro* suicide of TK-cAd-MSCs treated with or without GCV (0.002 μg/ μL) after 1, 5, 7 and 9 days (n = 6/each condition). Values represent means ± SD from three independent assays. Significant differences were considered when *P< 0.05 by t-test comparison.

cAd-MSCs were successfully transduced with CMV:hRluc:mRFP:tTK lentiviral particles (TK-cAd-MSCs). Cells were expanded and RFP expressing cells were sorted by FACS, fluorescence was later analyzed by fluorescent microscopy (Fig 1A−1C). BLI signal of TK-cAd-MSCs presented a linear correlation with cell number and allowed the detection of less than 40 cells (Fig 1D). TK-cAd-MSCs were exposed to GCV (0.004 μg/μL) and cell viability was analyzed by measuring BLI signal on days 1, 5, 7, and 9 after treatment. On day 5, 50% of TK-cAd-MSCs were killed by suicide effect and after 9 days of exposition to GCV the figure rose to 99% (Fig 1E).

### TK-cAd-MSCs maintain the proliferation rate, the MSCs-phenotype and karyotype stability

Transduced cells maintained their proliferation capacity compared with cAd-MSCs (S1A Fig). TK-cAd-MSCs present a similar surface expression of CD90, CD34, CD44, and CD105 MSCs markers (S1B Fig). Moreover, TK-transduction did not alter their chromosomal pattern (S2A and S2B Fig).

### TK-cAd-MSCs modified its secretory profile compared to cAd-MSCs

**Fig 2:**
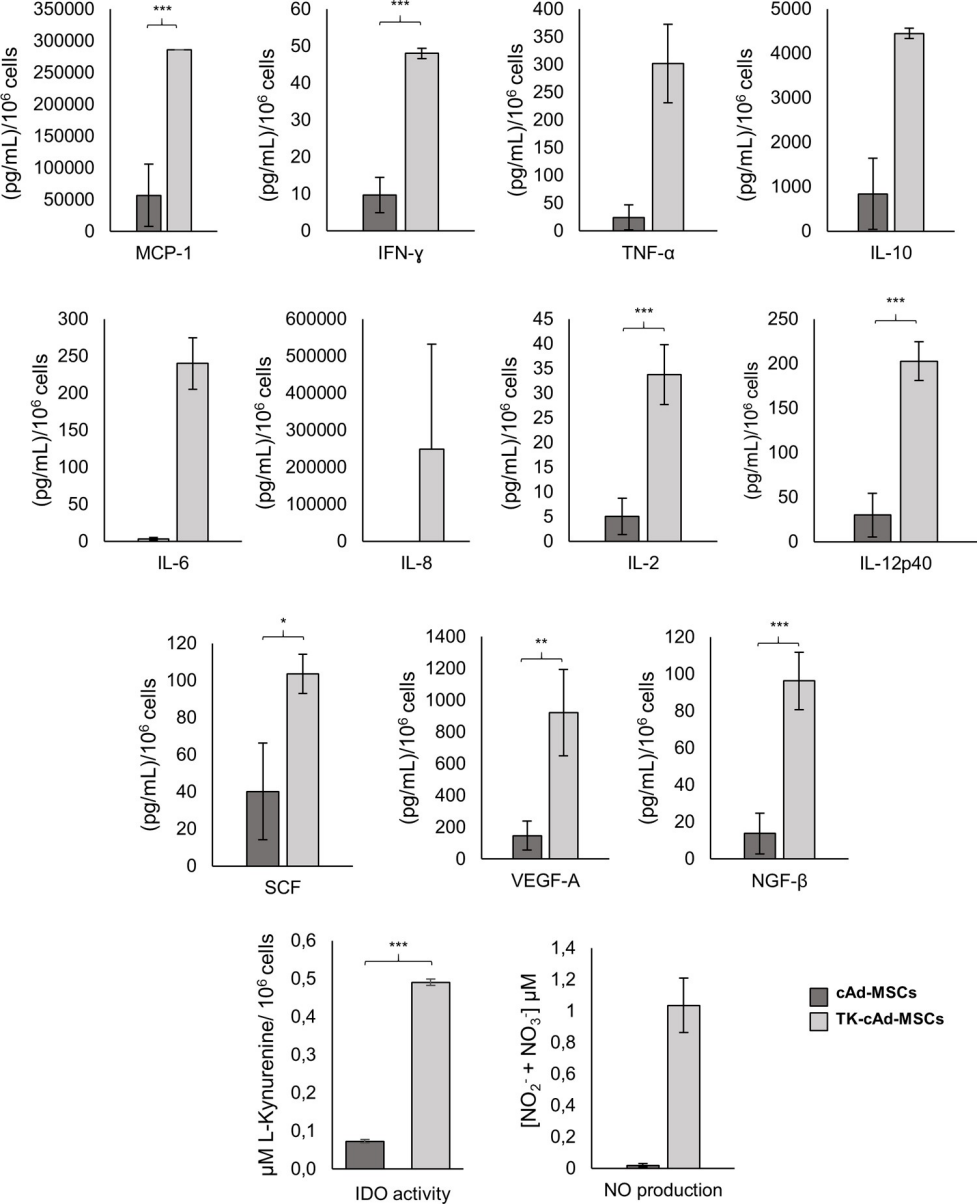
Secretory profile of cAd-MSCs and TK-cAd-MSCs. TK-cAD-MSCs present a higher expression of MCP-1, IFN-γ, IL-2, IL12p40 and VEGF-A including IDO enzymatic activity and NO production. Asterisks indicate significant differences between compared values P < 0.05 (*), P < 0.01 (**) and P < 0.001 (***).

TK-cAd-MSC significantly modified its secretory profile in some of the considered analytes in comparison to cAd-MSC (Fig 2). The ELISA kit showed a higher expression of MCP-1, IFN-γ, IL-2, IL12p40, and VEGF-A. The TK-cAd-MSC also showed a higher IDO enzymatic activity and NO production.

### TK-cAd-MSCs present higher exosome secretion and differences in their proteomic cargo compared to cAd-MSCs

**Fig 3:**
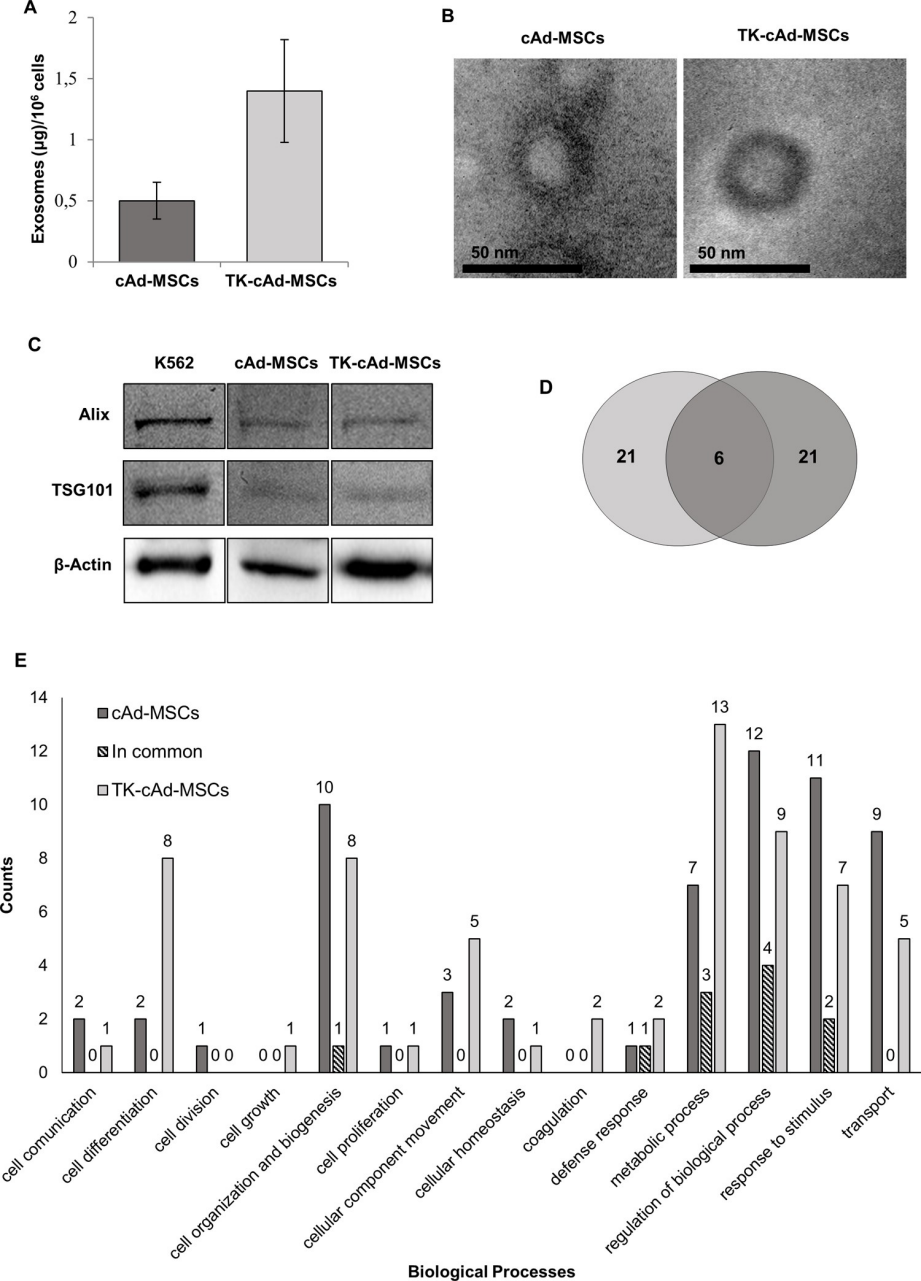
Exosome production of cAd-MSCs and TK-cAMSCs. (A) Exosome quantification. (B) Representative images of TEM. (C) Western blot analysis of ALIX and TSG101 exosomal markers in both cell types. (D) Venn diagram of cAd-MSCs and TK-cAd-MSCs exosomal proteins. (E) Exosomal protein distribution according to GO parameters.

TK-cAd-MSCs showed almost three times more amount of secreted exosomes than cAd-MSCs (Fig 3A). Both cell types’ exosomes showed a size distribution between 90−220 nm (S3 Fig); besides, vesicles of ~40 nm of diameter, compatible with exosome dimensions, were detectable by TEM (Fig 3B). Moreover, TK-cAd-MSCs and cAd-MSCs exosomes showed a similar positive expression in ALIX and TSG101 markers (Fig 3C).

Exosomal protein cargo was performed by mass spectrometry and analyzed using the *Canis lupus familiaris* protein database. We found 21 characterized proteins in TK-cAd-MSCs exosomes and 21 in cAd-MSCs exosomes, but only 6 proteins appeared in both exosomes (Fig 3D). Biological processes of characterized exosome proteins in both MSCs sources were determined by Gene Ontology (GO) parameters. TK-cAd-MSCs exosomes present a high level of proteins involved in cell differentiation, metabolic process, and coagulation. However, cAd-MSCs exosomal proteins are related to the regulation of biological processes, response to stimulus, and transport (Fig 3E). Proteins involved in cell death, conjugation, development, and reproduction were not found in any kind of cell. Information about the specific proteins involved in these different parameters is shown in the S1–S3 Tables. Proteomics and western blot analysis revealed that hRluc:mRFP:tTK protein construction did not appear in TK-cAd-MSCs exosome cargo without treatment with GCV. The secretome of TK-cAd-MSCs with and without GCV treatment was also evaluated and hRluc:mRFP:tTK protein construction was not found either. This fact indicates that hRluc:mRFP:tTK protein construction is not released into the culture medium by the cells.

### TK-cAd-MSCs demonstrate a significantly higher capacity for inhibition of cPBMCs in comparison to cAd-MSCs

**Fig 4:**
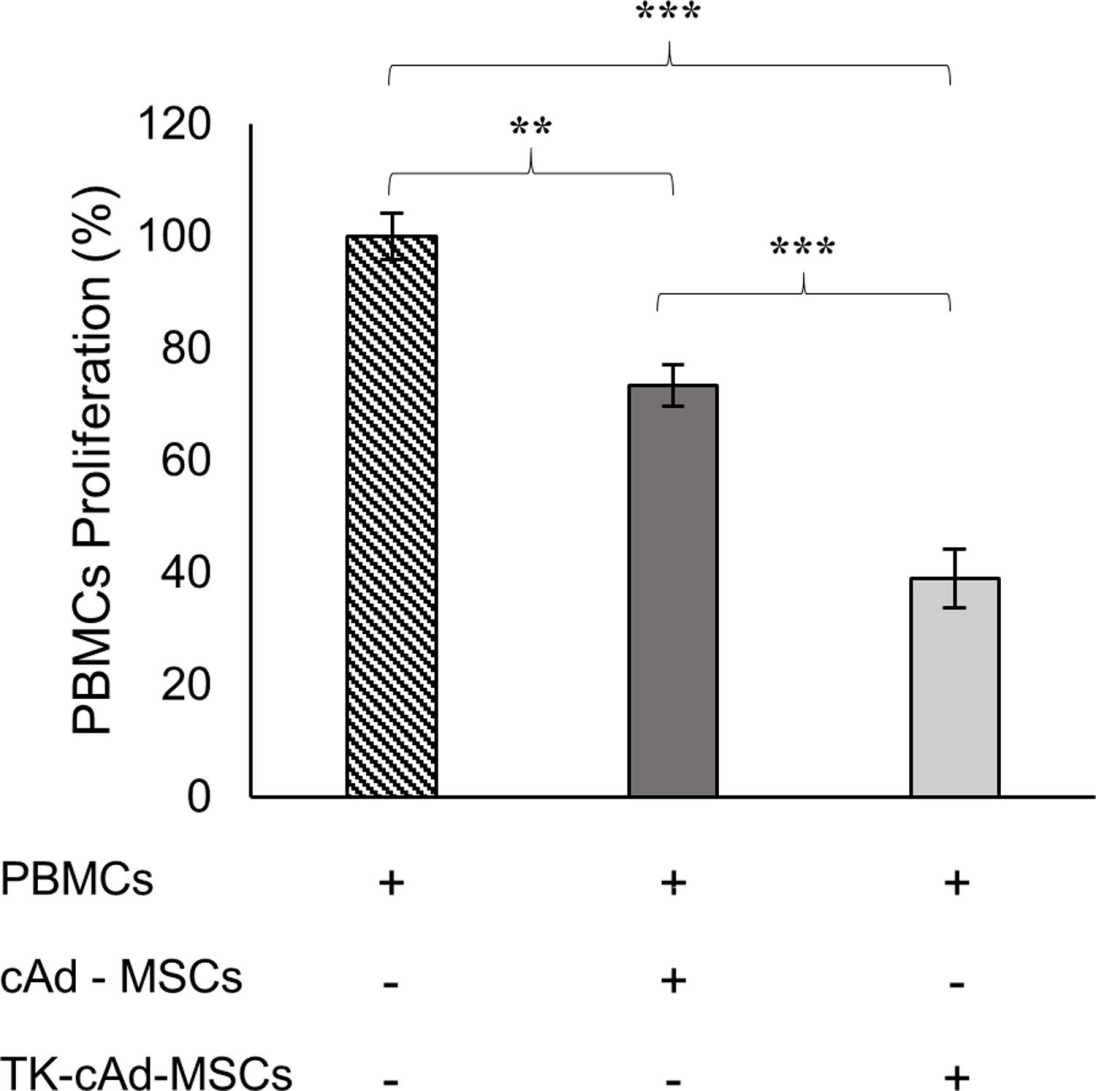
Immunomodulatory properties. Modulation of canine cPBMCs proliferation in presence of cAd-MSCs and TK-cAd-MSCs. Asterisks indicate significant differences between compared values P < 0.01 (**) and P < 0.001 (***).

When co-cultured with cPBMCs activated with ConA, both cell types exert a significant suppressive effect in cPBMCs proliferation, increasing significantly after transfection (Fig 4).

### TK-cAd-MSCs show antitumoral efficacy *in vitro*

**Fig 5:**
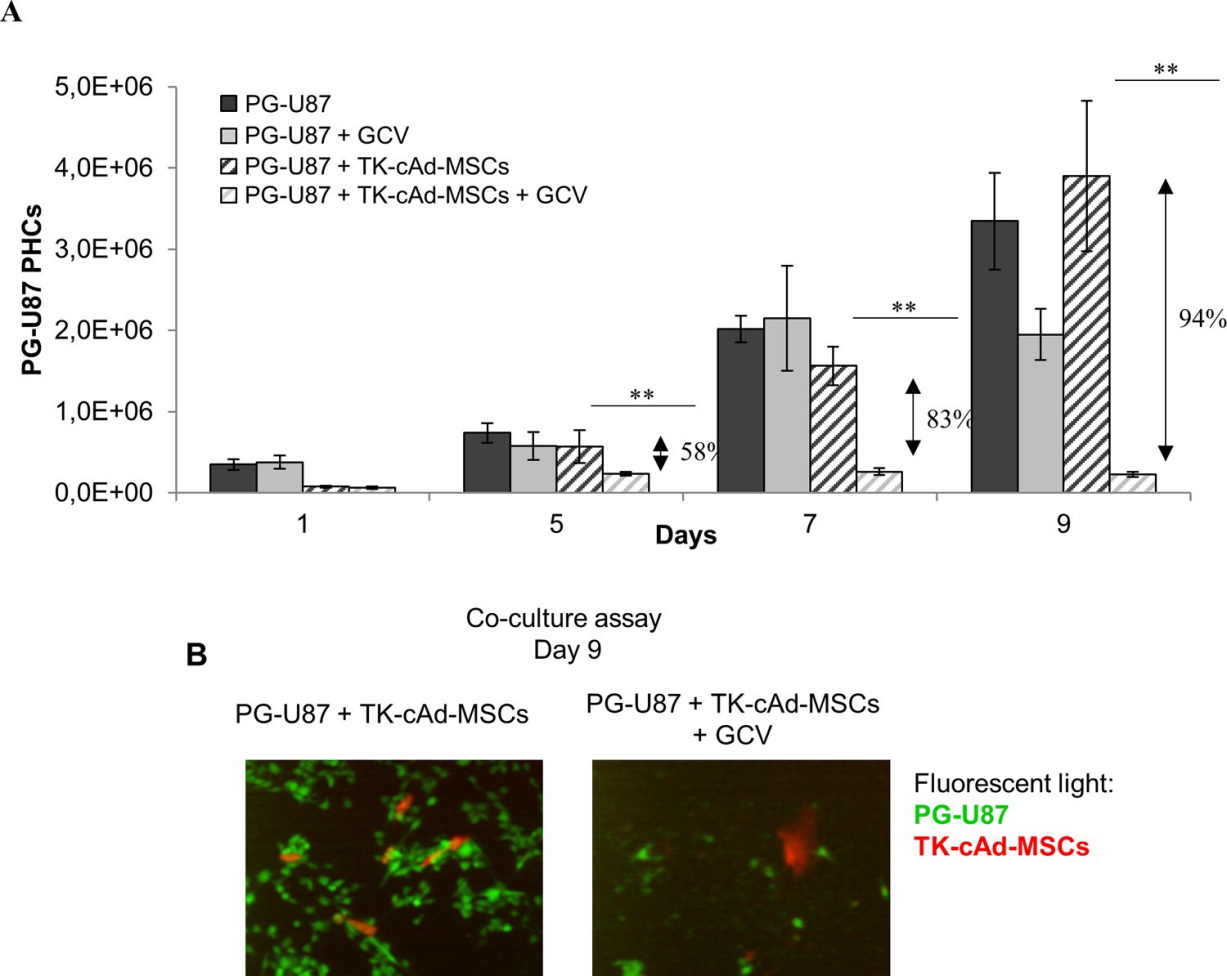
*In vitro* cytotoxicity of TK-cAd-MSCs against PG-U87 cells. (A) Histograms comparing the *in vitro* PG-U87 killing capacity of TK-cAd-MSCs co-cultured at 0:1 or 1:1 proportion of TK-cAd-MSCs:PG-U87 cells (n = 6/each condition). Values represent means ± SD from three independent assays. The percentage of proliferation inhibition by GCV treatment is indicated over the corresponding pair of bars. Statistically significant differences were considered when **P< 0.01, comparing non-treated *vs*. treated PG-U87 cells in each specific co-culture condition by two-way ANOVA test and Bonferroni post-test. (B) Fluorescence microscopy images of TK-cAd-MSCs (red) and PG-U87 cells (green) in a 1:1 co-culture ratio after 9 days, receiving or not GCV treatment. Microscope images were taken with a Nikon eclipse ts100 microscope, 10× objective.

Quantification of luciferase activity (Rluc) by BLI showed significant (p<0.01) antitumoral effects of TK-cAd-MSCs and bystander killing effect over co-cultured PG-U87 cells (Fig 5A). On day 9, fluorescence microscope and BLI images of representative cell culture wells showed a high confluence of PG-U87 cells interacting with TK-cAd-MSCS and emitting high-intensity light in comparison with the cells receiving GCV (0.004 mg/mL) (Fig 5B).

### TK-cAd-MSCs showed tumor cell killing capacity and significantly increased animal survival in a mice glioblastoma model

**Fig 6:**
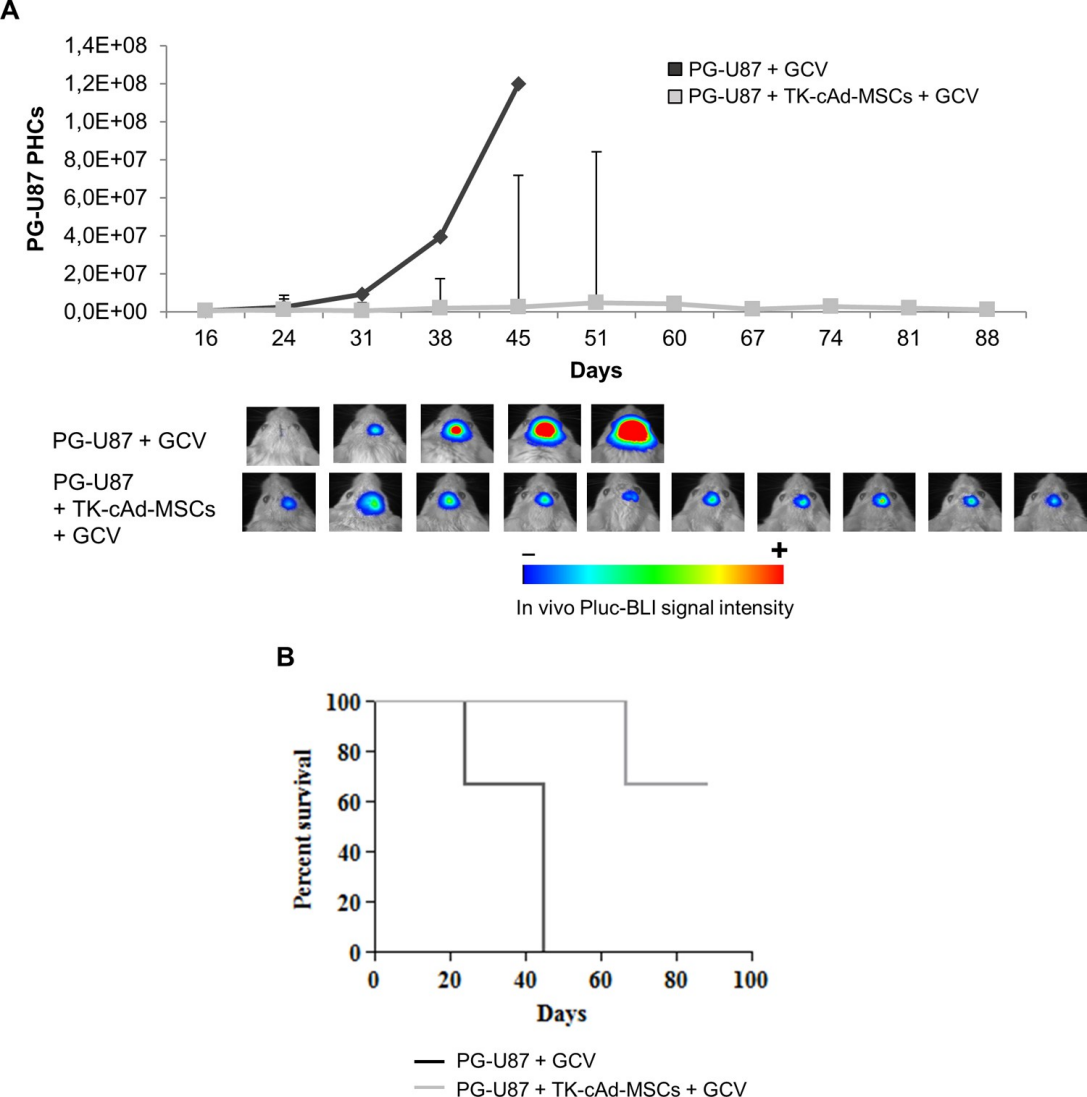
Effect of TK-cAd-MSCs against PG-U87 cells in an *in vivo* mouse model. (A) *In vivo* changes in light production by PG-U87 tumors expressed by the Median ± IQR of Pluc-BLI PHCs. No statistically significant differences between groups were obtained by the two-sample Wilcoxon rank-sum test. Insets, representative BLI images of PG-U87 tumor-bearing mice: top, control treated with GCV; bottom, treated with TK-cAd-MSCs plus GCV (n = 4 mice/group). BLI images were superimposed on black and white images. Arbitrary rainbow color scale depicts light intensity (red: highest; blue: lowest). (B) Kaplan-Meier plot showing animal survival throughout the experiment. Significant differences (*P< 0.05) were found by the Log-rank test.

Animal health and behaviour were monitored every day, and every ten days, mice were monitored to image luciferase activity by BLI (Fig 6A). Plots of light emitted by the PG-U87 cells showed that the control group has a tumor growth that increased progressively until day 40, on which all the animals had died due to the tumor. On the other hand, in mice treated with TK-cAd-MSCs, light production by tumor cells remained at a significantly lower level compared to control animals, indicating that tumor growth was inhibited. Kaplan-Meier plots showed that TK-cAd-MSCs therapy increased significantly (*P*<0.05) the survival in treated animals, in which some survived 90 days (Fig 6B). At this point, the remaining animals alive were euthanized for ethical reasons.

## Discussion

Gliomas are a therapeutic challenge for both human and canine species, representing the most common form of malignant primary brain tumors in humans and the second most common form in dogs [24].

In the last decade, there has been great interest in MSCs genetic modification with suicidal genes to meet therapeutic cytotoxic effect against cancer [3, 4, 13].

Gene-directed enzyme/prodrug therapy using Ad-MSCs expressing TK has proven to be a promising alternative in glioblastoma therapy [10, 11, 23, 25].

TK-Ad-MSCs have the capacity to migrate and home into the tumor vasculature and then exert selective and localized cytotoxicity against tumor cells [23]. Therefore, it is essential to ensure that the modification of the MSCs does not induce aberrations in their behavior and is safe.

In our study, we transduced cAd-MSCs for the expression of TK, aiming to characterize cellular aspects of TK-cAd-MSCs and compare them with the original non-transduced cAd-MSCs, assessing for the first-time proliferation, phenotypic expression, chromosomal stability, secretory profile, immunomodulatory potential, and antitumor efficacy *in vitro* and *in vivo* in a mice model with the glioblastoma cell line U87.

By transfection using lentivirus, we managed to produce chromosomally stable TK-cAd-MSCs, maintain their proliferation rate and the expression of MSCs markers, increasing the expression of CD44 and CD105.

Genetic modification significantly affects its secretory profile, both the soluble factors analyzed and the fraction of exosomes. TK-cAd-MSCs showed a high secretory profile of some active antitumor immune response cytokines as IFN-γ, IL-2, and chemokines as MCP-1, IL12p40. IFN-γ has potent antitumor effects in the tumor microenvironment [26]. IL-2 is one of the key cytokines with pleiotropic effects on the immune system and antitumor activity, and its use is approved for cancer immunotherapy [27, 28].

Chemokines are produced as a response to the pro-inflammatory stimulus to attract and activate different effector immune cells. MCP-1 is a potent monocyte-attracting chemokine and contributes greatly to the recruitment of blood monocytes, neutrophils, and NKT cells at sites where inflammatory responses and tumors are taking place [29]. IL-12p40 has an important role in the development of T cells while enhancing the production of immune factors and contributing to the immune response in the virus infection of severe disease [30, 31].

We observed a threefold increase in the number of exosomes secreted by the modified cells, with a much more homogeneous size compared to the cAd-MSCs. Transfection also determines a change in the proteomic profile of its cargo, with abundant proteins that participate in biological processes related to cell mobilization, coagulation, defense response, and metabolic processes. We have found only 6 proteins shared between both cell types such as Annexin, Annexin A2, Keratin, type II cytoskeletal 1 and 5, Milk fat globule-EGF factor 8 protein, and Ubiquitin-60S ribosomal protein L40.

Considering our results, we believe that more experiments would be necessary to demonstrate that the secretome or some of its components, such as exosomes, may be involved in the mechanism of action of these cells, corroborating what has been described with human MSCs [32].

We found that when GCV is added to TK-cAd-MSCs, they have cytotoxic capacity against U87 cells *in vitro* and *in vivo* mice models, obtaining levels of tumor cell death comparable to previous literature [10, 14]. The use of a glioblastoma cell line U87 to assess its antitumor capacity is justified since it is the most used model in our studies [6, 7, 10, 23]. In this model, we observed a significant increase (more than twice) in the survival of the animals treated with TK-cAd-MSCs plus GCV. New studies on canine tumor cell lines would be necessary to confirm their antitumor efficacy before proposing these transfected cells as a therapeutic tool for cancer in dogs.

The advantage of our approach is that the TK-cAd-MSCs could be effectively implanted locally in the tumor area so that subsequently the GCV could be administered systemically. With this protocol, the cytotoxic product is produced exclusively from the GCV by the TK-cAd-MSCs and, consequently, through the bystander mechanism, the tumor cells will be affected when they acquire sensitivity to the GCV [10, 14]. The bystander effect, which involves the propagation of the cytotoxic molecules of phosphorylated GCV to adjacent non-transduced tumor cells, has been attributed to possible different pathways, although this mechanism is not fully explained [4, 13]. In order to elucidate this mechanism, proteomics and western blot analysis revealed that hRluc:mRFP:tTK protein construction did not appear in TK-cAd-MSCs exosome cargo without GCV treatment. TK-cAd-MSCs secretome with and without GCV treatment was also assessed and hRluc:mRFP:tTK protein construction was not found either. This fact indicates that hRluc:mRFP:tTK protein construction is not released directly into the culture medium by the cells, therefore other mechanisms must be involved.

Our work presents some limitations, such as the use of a U87 human glioblastoma cell line with an in vivo murine model. However, we believe that our study provides new perspectives in the knowledge of transfected canine MSCs, the antitumor potential of the gene therapy in canine glioma and provides information for future clinical studies of safety and efficacy in canine oncologic patients, with the idea of translating this knowledge to possible application in humans [1].

In summary, we demonstrate the potential to genetically modify cAd-MSCs with a lentiviral vector efficiently to express the herpes simplex virus TK and that in combination with GCV prodrug demonstrated effective antitumor potential on U-87 glioblastoma cell line in vitro and in vivo mice model.

TK-cAd-MSCs maintained cell proliferation, karyotype stability, and MSCs phenotype. Additionally, genetic modification affects its secretory profile, increasing significantly some antitumor immune soluble factors as IFN-γ, IL-2, MCP-1, and IL12p40 and increases the production of exosomes with a change in the proteomic profile of its cargo.

## Supporting information

S1 Fig cAd-MSCs and TK-cAd-MSCs proliferation and phenotype characterization. (A) Cell proliferation was performed by MTS assay. (B) Surface expression analysis of CD90, CD34, CD44, and CD105 MSCs markers in TK-cAd-MSCs and cAd-MSCs.
(PDF)

S2 Fig Karyotype. cAd-MSCs (A) and TK-cAd-MSCs (B) karyotypes.
(PDF)

S3 Fig Size distribution, polydispersity index, and zeta potential (electronegativity) (mV) of cAd-MSCs and TK-cAd-MSCs exosomes. (PDF)

S1 Raw images (PDF)

S1 Table List of specific proteins in cAd-MSCs exosomes. Proteomic analysis parameters such as accession to Uniprot protein database, molecular weight (MW), scores, number of peptides, and coverage are shown. Biological Functions are indicated according to Gene Ontology parameters.
(PDF)

S2 Table List of specifics proteins in TK-cAd-MSCs exosomes. Proteomic analysis parameters such as accession to Uniprot protein database, molecular weight (MW), scores, number of peptides, and coverage are shown. Biological Functions are indicated according to Gene Ontology parameters.
(PDF)

S3 Table List of specific proteins in common present in exosomes of both cell types. Proteomic analysis parameters such as accession to *Uniprot* protein database, molecular weight (MW), scores, number of peptides and coverage are shown. Biological Functions are indicated according to *Gene Ontology* parameters.
(PDF)
