# Supplementary figures and images for: Suicide gene therapy by canine mesenchymal stem cell transduced with thymidine kinase in a u-87 glioblastoma murine model: Secretory profile and antitumor activity
Source: PLoS One. 2022 Feb 15;17(2):e0264001. doi: 10.1371/journal.pone.0264001 (PMC8846542; doi:10.1371/journal.pone.0264001)

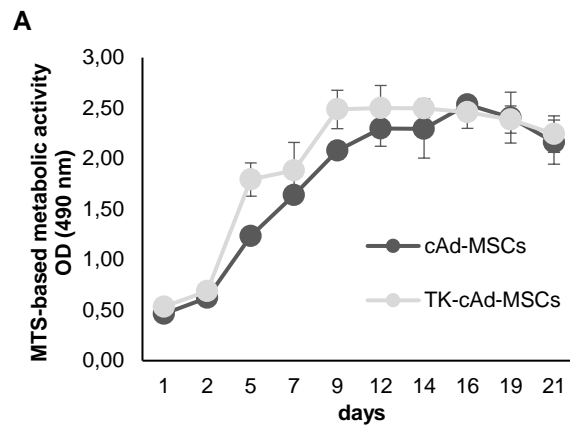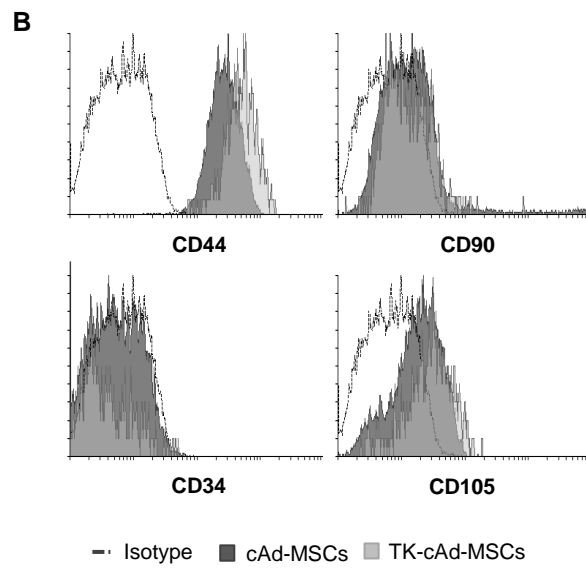

Supplement: S1 Fig — (A) Cell proliferation was performed by MTS assay. (B) Surface expression analysis of CD90, CD34, CD44, and CD105 MSCs markers in TK-cAd-MSCs and cAd-MSCs. (PDF) [file pone.0264001.s001.pdf]

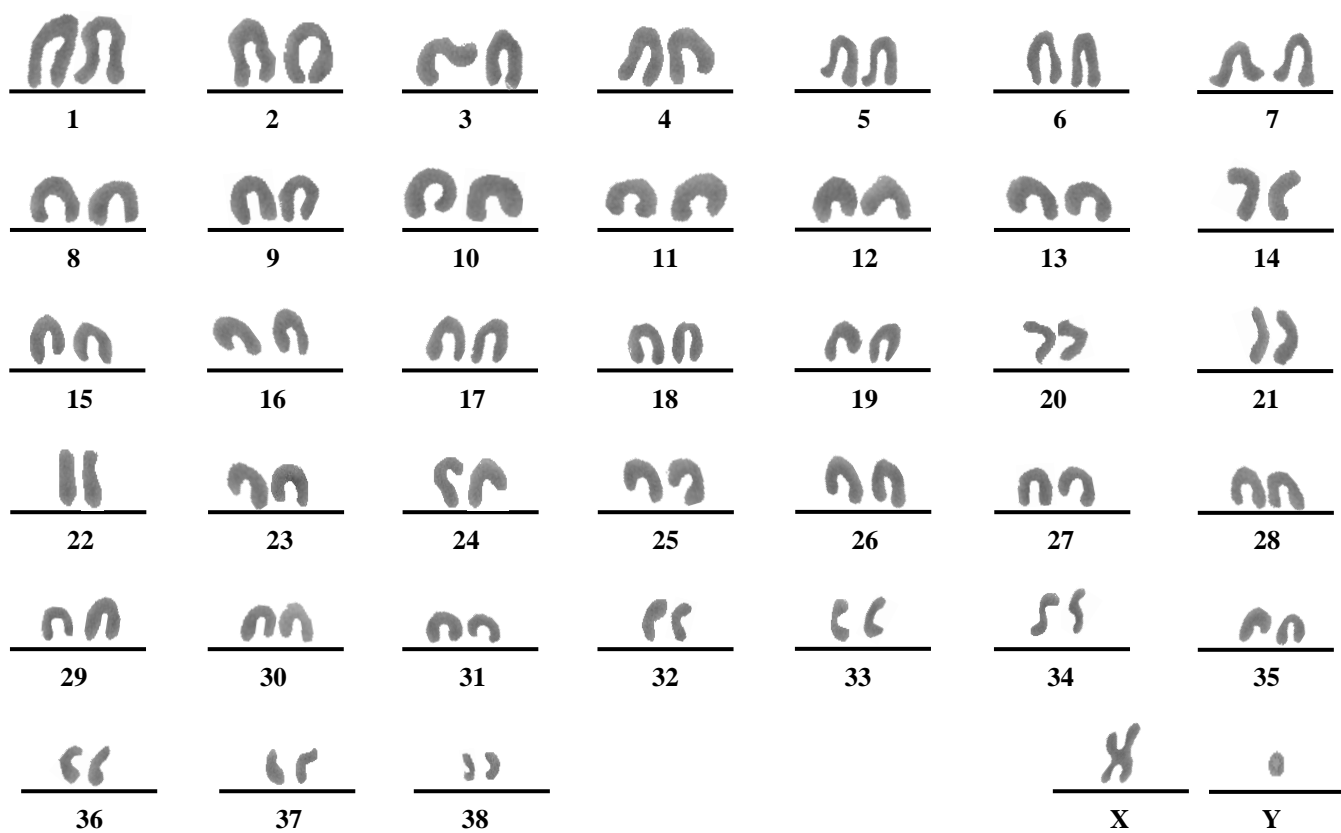

*cAd-MSCs* Chromosomes

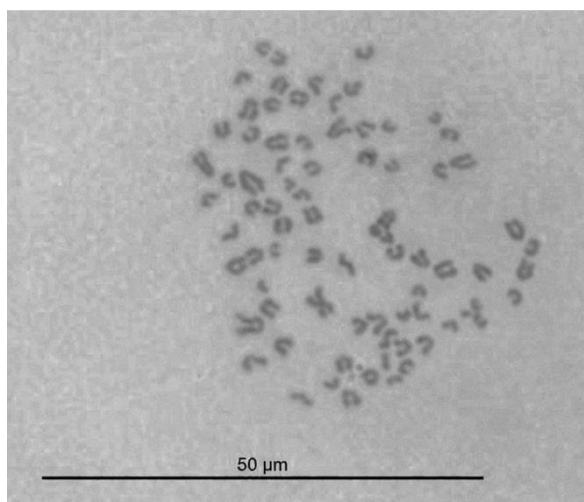

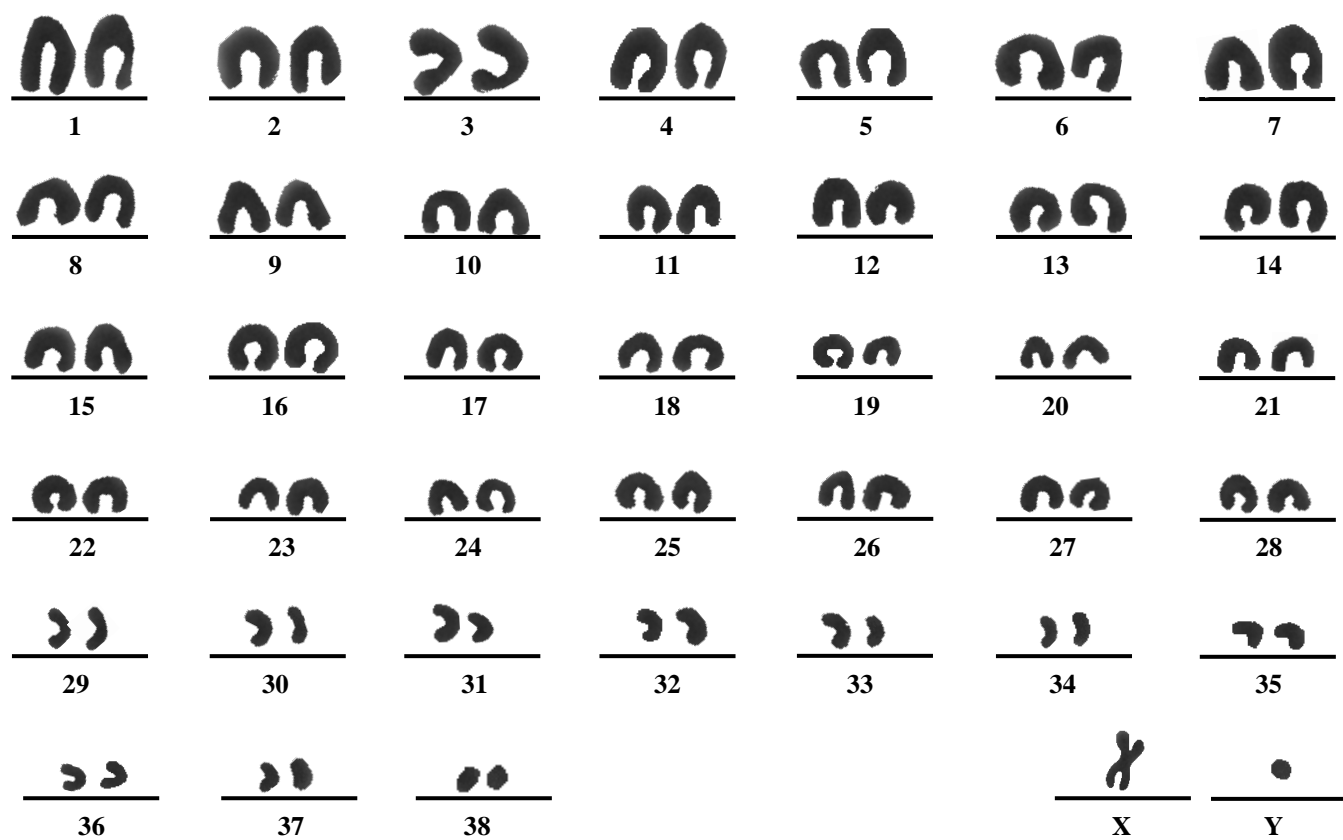

*TK-cAd-MSCs* Chromosomes

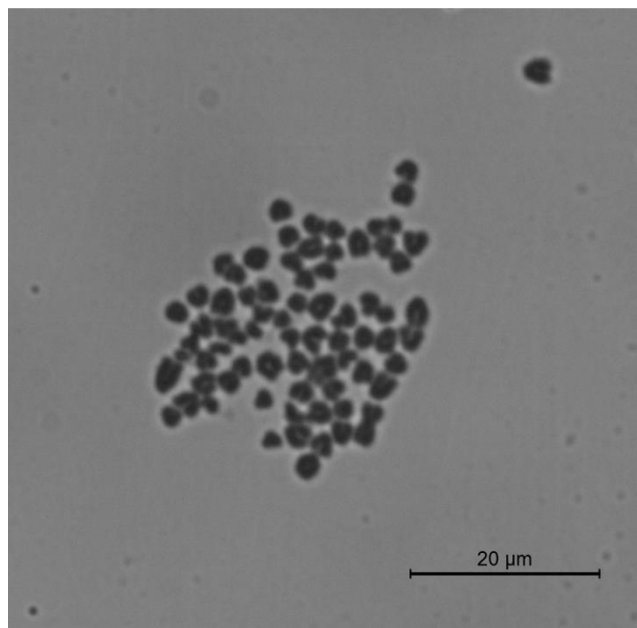

Supplement: S2 Fig — cAd-MSCs (A) and TK-cAd-MSCs (B) karyotypes. (PDF) [file pone.0264001.s002.pdf]

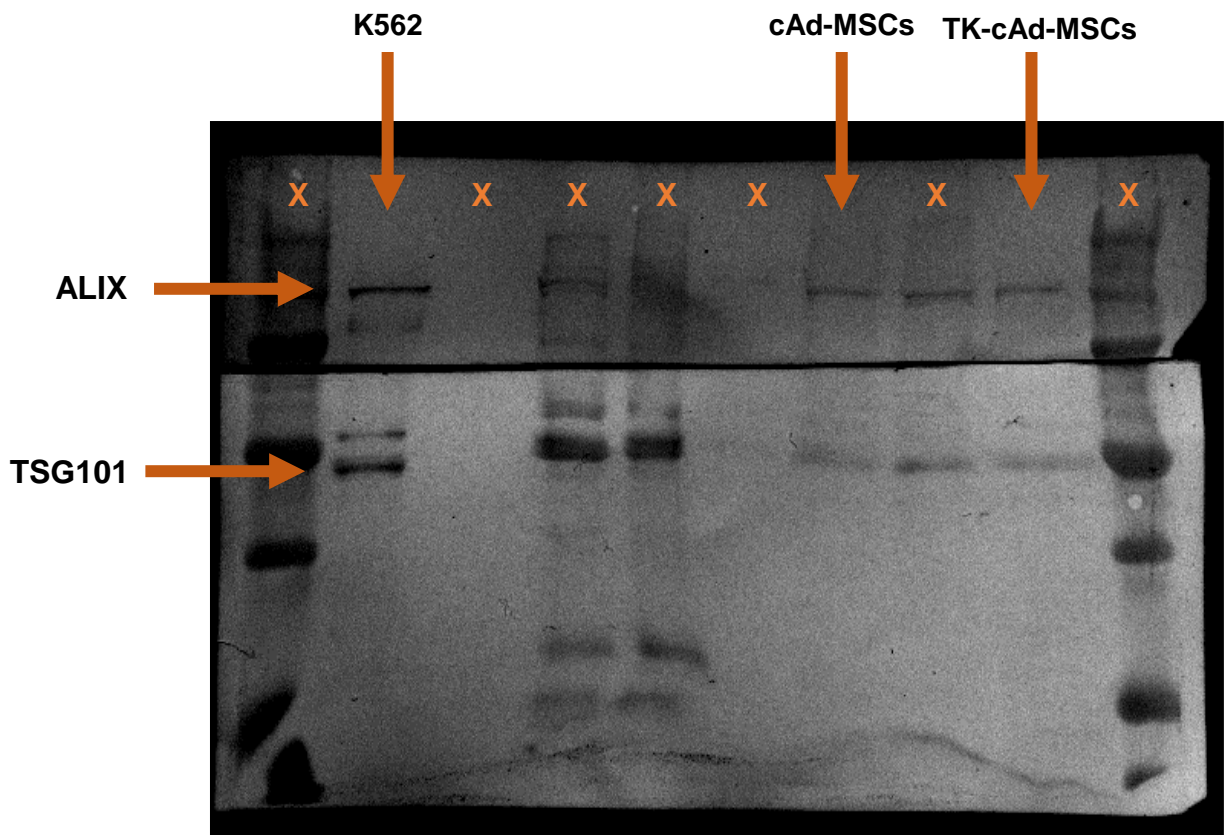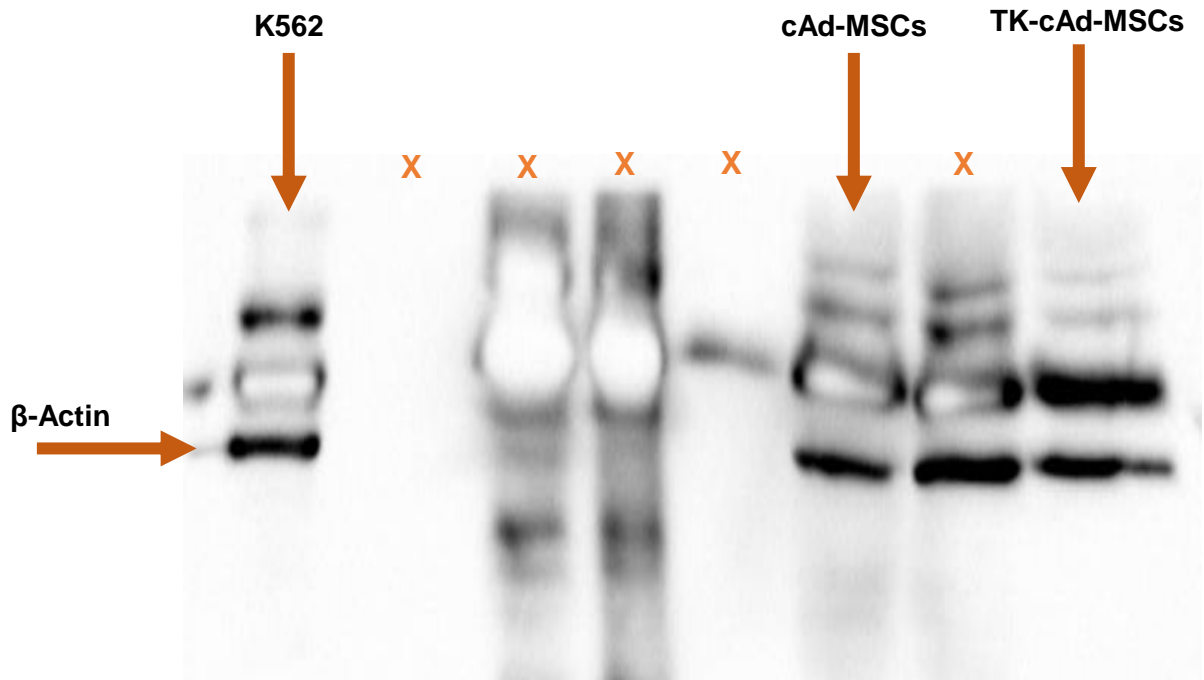

Supplement: S1 Raw images — (PDF) [file pone.0264001.s004.pdf]
