# Supplementary material for: Suicide gene therapy by canine mesenchymal stem cell transduced with thymidine kinase in a u-87 glioblastoma murine model: Secretory profile and antitumor activity
Source: PLoS One. 2022 Feb 15;17(2):e0264001. doi: 10.1371/journal.pone.0264001 (PMC8846542; doi:10.1371/journal.pone.0264001)

	Polydispersity Index (PDI)	Zeta potential (Electronegativity) (mV)	Temperature (ºC)
cAd-MSCs-Exosomes	0.544	-13,2 ± 0.4	25
TK-cAd-MSCs-Exosomes	0.363	-13,3 ± 0.9	25

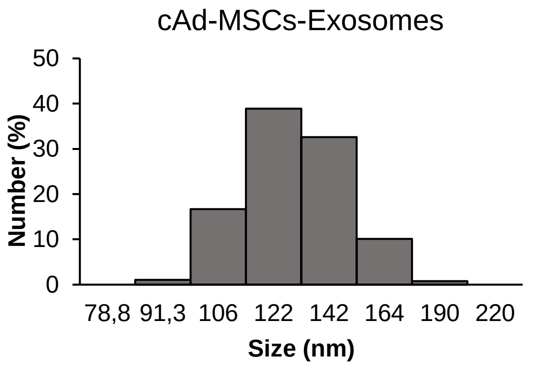

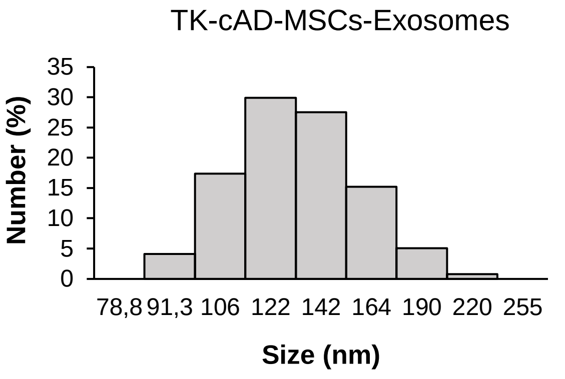

Supplement: S3 Fig — (PDF) [file pone.0264001.s003.pdf]
