# Supplementary material for: Suicide gene therapy by canine mesenchymal stem cell transduced with thymidine kinase in a u-87 glioblastoma murine model: Secretory profile and antitumor activity
Source: PLoS One. 2022 Feb 15;17(2):e0264001. doi: 10.1371/journal.pone.0264001 (PMC8846542; doi:10.1371/journal.pone.0264001)
Supplement: S1 Table — Proteomic analysis parameters such as accession to Uniprot protein database, molecular weight (MW), scores, number of peptides, and coverage are shown. Biological Functions are indicated according to Gene Ontology parameters. (PDF) [file pone.0264001.s005.pdf]

Number	Accession	Protein	MW [kDa]	Score	Peptides	Coverage [%]	Biological Functions
1	ACTB_CANLF	Actin, cytoplasmic 1	41.7	831.7	15	50.9	Cell organization and biogenesis
2	K1C10_CANLF	Keratin, type I cytoskeletal 10	57.7	464.7	9	14.6	Cell organization and biogenesis
3	F1PG82_CANLF	ATP-binding cassette sub-family G member 2	73.0	250.2	4	7.0	Metabolic process<br>Transport
4	RAB1A_CANLF	Ras-related protein Rab-1A	22.7	226.9	3	21.5	Cell organization and biogenesis<br>Cellular component movement<br>Defence response<br>Metabolic process<br>Regulation of biological process<br>Response to stimulus<br>Transport
5	E2QW85_CANLF	Elongation factor 1-alpha	49.9	208.4	4	11.5	Metabolic process
6	F2Z4Q6_CANLF	Serum albumin	68.6	180.8	4	5.8	Cell communication<br>Regulation of biological process<br>Response to stimulus<br>Transport

7	E2QSF4_CANLF	Tubulin beta chain	49.6	175.2	5	15.5	Cell organization and biogenesis<br>Cellular component movement
8	F1PZA1_CANLF	Isocitrate dehydrogenase [NADP]	46.8	170.5	3	9.9	Metabolic process<br>Regulation of biological process<br>Response to stimulus
9	L7N0G4_CANLF	Tubulin alpha chain	50.1	144.5	2	7.3	Cell organization and biogenesis
10	RAB7A_CANLF	Ras-related protein Rab-7a	23.5	139.1	2	13.0	Cell organization and biogenesis<br>Cellular homeostasis<br>Metabolic process<br>Regulation of biological process<br>Response to stimulus<br>Transport
11	GNAI2_CANLF	Guanine nucleotide-binding protein G(i) subunit alpha-2	40.5	134.7	3	11.8	Cell division<br>Cell proliferation<br>Regulation of biological process<br>Response to stimulus
12	F2Z4P9_CANLF	Ras-related protein Rab-10	22.5	117.4	2	11.5	Cell differentiation<br>Cell organization and biogenesis<br>Regulation of biological process<br>Response to stimulus<br>Transport
13	F1Q0Q9_CANLF	Keratin 17	48.1	116.6	3	7.2	Cell organization and biogenesis<br>Regulation of biological process<br>Response to stimulus

14	F1PK62_CANLF	Peptidyl-prolyl cis-trans isomerase	18.0	113.6	2	7.9	Metabolic process
15	RAC1_CANLF	Ras-related C3 botulinum toxin substrate 1	21.4	104.2	3	18.2	Cellular component movement<br>Regulation of biological process<br>Response to stimulus
16	E2RF35_CANLF	Calcium-transporting ATPase	136.7	103.3	2	2.3	Cell differentiation<br>Cellular homeostasis<br>Transport
17	F1PGZ8_CANLF	G protein-coupled receptor class C group 5 member B	43.0	90.5	2	6.2	Regulation of biological process<br>Response to stimulus
18	J9P7X9_CANLF	Gap junction protein	43.0	86.3	1	3.7	Cell communication<br>Regulation of biological process<br>Response to stimulus<br>Transport
19	F1PSK6_CANLF	EH domain containing 1	53.1	85.9	2	5.5	Cell organization and biogenesis<br>Regulation of biological process<br>Transport
20	J9P2B7_CANLF	Histone H2A	13.9	83.3	2	21.9	Regulation of biological process
21	J9P843_CANLF	Apolipoprotein A-I	15.7	80.5	2	21.0	Metabolic process<br>Transport

S1 Table. List of specific proteins in cAd-MSCs exosomes. Proteomic analysis parameters such as accession to *Uniprot* protein database, molecular weight (MW), scores, number of peptides and coverage are shown. Biological Functions are indicated according *Gene Ontology* parameters.
