# Supplementary material for: Suicide gene therapy by canine mesenchymal stem cell transduced with thymidine kinase in a u-87 glioblastoma murine model: Secretory profile and antitumor activity
Source: PLoS One. 2022 Feb 15;17(2):e0264001. doi: 10.1371/journal.pone.0264001 (PMC8846542; doi:10.1371/journal.pone.0264001)
Supplement: S2 Table — Proteomic analysis parameters such as accession to Uniprot protein database, molecular weight (MW), scores, number of peptides, and coverage are shown. Biological Functions are indicated according to Gene Ontology parameters. (PDF) [file pone.0264001.s006.pdf]

Number	Accession	Protein	MW [kDa]	Scores	Peptides	Coverage [%]	Biological Processes
1	F1PYU9_CANLF	Keratin, type I cytoskeletal 10	57.7	744.2	14	17.1	Cell differentiation<br>Metabolic process
2	J9NXE2_CANLF	Actin, cytoplasmic 1	41.2	648.6	16	41.8	Cell organization and biogenesis
3	F6UME0_CANLF	Alpha-2-macroglobulin	165.1	479.3	10	5.8	Cell differentiation<br>Regulation of biological process
4	F1PTX4_CANLF	Keratin, type II cytoskeletal 2 epidermal	64.7	457.6	10	10.4	Cell organization and biogenesis<br>Cell proliferation<br>Cellular component movement<br>Metabolic process
5	J9P8M2_CANLF	Fibronectin	249.0	429	11	4.2	Defence response<br>Regulation of biological process<br>Response to stimulus
6	ALBU_CANLF	Serum albumin	68.6	348.8	7	9.4	Cell communication<br>Regulation of biological process<br>Response to stimulus<br>Transport
7	F1Q0R0_CANLF	Keratin 14	52.3	312	5	7.5	Cell differentiation<br>Cell organization and biogenesis
8	MYH9_CANLF	Myosin-9	226.3	244.8	7	2.9	Cell differentiation<br>Cell organization and biogenesis<br>Cellular component movement<br>Metabolic process

							Regulation of biological process<br>Transport
9	J9NU04_CANLF	Elongation factor 1-alpha	49.9	228.3	4	7.4	Metabolic process
10	FETA_CANLF	Alpha-fetoprotein	68.7	206.6	7	8.5	Transport
11	E2REU6_CANLF	Keratin, type I cytoskeletal 18	49.8	179	5	8.3	Cell differentiation<br>Metabolic process
12	F1PHR2_CANLF	Pyruvate kinase	59.6	177.8	4	5.9	Metabolic process<br>Response to stimulus
13	APOA1_CANLF	Apolipoprotein A-I	30.2	173	5	11.7	Cell organization and biogenesis<br>Metabolic process<br>Regulation of biological process<br>Response to stimulus<br>Transport
14	CO1A1_CANLF	Collagen alpha-1(I) chain	138.7	143.6	4	3.0	Cell organization and biogenesis
15	F1PTZ9_CANLF	Glyceraldehyde-3-phosphate dehydrogenase	35.5	138.9	5	15.2	Metabolic process
16	F1PW98_CANLF	Keratin, type II cytoskeletal 8	55.0	129.9	3	5.1	Metabolic process

17	F1PW65_CANLF	Fibrinogen beta chain	56.3	123.9	3	4.6	Cell organization and biogenesis<br>Coagulation<br>Defence response<br>Metabolic process<br>Regulation of biological process<br>Response to stimulus
18	F1Q421_CANLF	Plasminogen	91.1	116.4	2	1.4	Cell differentiation<br>Cellular component movement<br>Cellular homeostasis<br>Coagulation<br>Metabolic process<br>Regulation of biological process
19	E2RT60_CANLF	Integrin beta	88.1	113.4	4	5.3	Cell differentiation<br>Cell growth<br>Cell organization and biogenesis<br>Cellular component movement<br>Metabolic process<br>Regulation of biological process<br>Response to stimulus<br>Transport
20	F1PIT4_CANLF	Tetraspanin	25.6	111.9	3	10.5	Cell differentiation<br>Cellular component movement<br>Regulation of biological process<br>Response to stimulus<br>Transport
21	J9NWS3_CANLF	Keratin, type I cytoskeletal 9	59.4	100.1	2	2.7	Metabolic process

S2 Table. List of specifics proteins in TK-cAd-MSCs exosomes. Proteomic analysis parameters such as accession to *Uniprot* protein database, molecular weight (MW), scores, number of peptides and coverage are shown. Biological Functions are indicated according *Gene Ontology* parameters.
