# Supplementary material for: Suicide gene therapy by canine mesenchymal stem cell transduced with thymidine kinase in a u-87 glioblastoma murine model: Secretory profile and antitumor activity
Source: PLoS One. 2022 Feb 15;17(2):e0264001. doi: 10.1371/journal.pone.0264001 (PMC8846542; doi:10.1371/journal.pone.0264001)
Supplement: S3 Table — Proteomic analysis parameters such as accession to Uniprot protein database, molecular weight (MW), scores, number of peptides and coverage are shown. Biological Functions are indicated according to Gene Ontology parameters. (PDF) [file pone.0264001.s007.pdf]

Number	Accession	Protein	MW [kDa]	Scores	Peptides	Coverage [%]	Biological Functions
1	ANXA2_CANLF	Annexin A2	38.6	408	8	24.8	Regulation of biological process
2	F1PTY1_CANLF	Keratin, type II cytoskeletal 1	63.7	346.2	6	10.8	Defence response<br>Metabolic process<br>Regulation of biological process<br>Response to stimulus
3	F1PFZ5_CANLF	Milk fat globule-EGF factor 8 protein	47.8	328.4	5	14.0	Cell organization and biogenesis<br>Regulation of biological process
4	RL40_CANLF	Ubiquitin-60S ribosomal protein L40	14.7	221.6	3	29.7	Metabolic process
5	E2R8Z5_CANLF	Keratin, type II cytoskeletal 5	62.7	153.3	3	5.7	Metabolic process
6	E2RQ14_CANLF	Annexin	35.9	110.3	2	8.4	Regulation of biological process<br>Response to stimulus

S3 Table. List of specific proteins in common present in exosomes of both cell types. Proteomic analysis parameters such as accession to *Uniprot* protein database, molecular weight (MW), scores, number of peptides and coverage are shown. Biological Functions are indicated according *Gene Ontology* parameters.
